# Heavy Metal Pollution and Assessment of the Bioaccumulation Potential of Earthworms from the Soil of Punjab, Pakistan

**DOI:** 10.3390/biology15040306

**Published:** 2026-02-09

**Authors:** Ting Shen, Javaria Altaf, Ghulam Abbas, Muhammad Naeem, Maryam Riasat, Aqsa Sarwar, Rabiya Hussain, Aqsa Faisal, Areej Fatima, Nawaz Haider Bashir, Huanhuan Chen

**Affiliations:** 1 College of Biology and Food Engineering, Qujing Normal University, Qujing 655011, China; shenting@mail.kib.ac.cn (T.S.); naeem@mail.qjnu.edu.cn (M.N.);; 2 Yunnan International Joint Laboratory with South and Southeast Asia for the Integrated Development of Animal-Derived Anti-Thrombosis Chinese Medicine, Qujing Normal University, Qujing 655011, China; 3 Department of Zoology, Government College University Faisalabad, Faisalabad 38000, Punjab, Pakistan; 4 Institute of Molecular Biosciences, The University of Queensland, Brisbane 4072, Australia; 5 Department of Zoology, Faculty of Engineering and Applied Sciences, Riphah International University, Faisalabad Campus, Faisalabad 44000, Punjab, Pakistan; 6 Faculty of Life Sciences, Beaconhouse College Programme, Faisalabad 38000, Punjab, Pakistan

**Keywords:** earthworms, Oligochaeta, heavy metals, bioaccumulation, pollution index, Central Punjab

## Abstract

Earthworms are widely used as biological indicators of soil contamination and are often referred to as the ‘intestine of the soil’; however, their effectiveness can vary across regions and environmental conditions. The performance of earthworms as indicators of soil pollution may vary among regions, and there is a need to assess their reliability under specific environmental conditions. So, in this study, soil and earthworm samples were collected from 19 locations across Punjab, Pakistan, to assess heavy metal pollution and bioaccumulation using geoaccumulation indices (Igeo) and bioaccumulation factors (BAF). Soil and earthworm tissues were digested and analyzed for heavy metals such as Sr, Zn, Ca, Cu, Fe, Mn, Co, Cr, Pb, Cd and Ni using ICP-MS. According to Igeo, cadmium frequently classified soils as strongly to extremely polluted, with values up to 4.5 in Bahawalnagar, whereas most other metals fell within the unpolluted to moderately polluted classes. Earthworms showed clear bioaccumulation (BAF > 1) for several essential or poorly regulated elements, particularly Ca, Mn, Cu and Sr; the maximum BAF for Ca reached 8.18 in Faisalabad. In contrast, group-1 carcinogenic metals (Pb, Cd and Cr) generally did not exhibit strong bioaccumulation. Relationships between BAF and soil metal concentrations or abiotic properties (moisture, pH, electrical conductivity, total dissolved and suspended solids) were generally weak (R^2^ typically ≤ 0.32), indicating that these factors explain only a modest fraction of the variation in metal uptake. Overall, the results highlight cadmium as the primary soil contamination risk in the surveyed areas. Because bioaccumulation showed weak relationships with soil metal concentrations and physicochemical parameters, earthworms in this study demonstrated limited effectiveness as bioindicators of toxic heavy metal accumulation. The limited internal accumulation of highly toxic metals suggests possible physiological regulation or tolerance, although the long-term ecological implications require further investigation. Further controlled studies are necessary to better evaluate their ecological role and potential applications in soil remediation, waste management, and vermicompost production.

## Simple Summary

The presence of heavy metals in soil can create a serious problem for soil life and can reduce agricultural productivity and have serious negative impacts on human health. Here, the soil contamination of Punjab, Pakistan, was assessed based on the contaminants of heavy metals and was assessed based on how much these heavy metals move into the earthworm’s body. This study assessed soil contamination across Punjab, Pakistan, and examined how toxic metals move from the soil into earthworms, which are commonly used as indicators of the soil condition, although their effectiveness can vary depending on the environmental conditions. Soil and earthworm samples were collected from nineteen different locations and tested for several metals, including cadmium, lead, copper, zinc, iron, and nickel. The findings revealed that cadmium posed the greatest risk, with many areas showing high to very high levels of contamination, while most other metals remained within safe limits. Earthworms were able to absorb certain metals from the soil, particularly calcium, nickel, manganese, strontium, and copper, although the degree of uptake varied among sites. However, they did not show strong accumulation of highly toxic metals such as cadmium and lead. Environmental factors such as soil moisture, pH, and salinity showed only weak relationships with metal uptake, indicating that these factors explained only a small portion of the variation. The study concludes that cadmium pollution represents a major threat to soil quality in Punjab. Because bioaccumulation showed weak relationships with soil metal concentrations and physicochemical properties, earthworms in this region demonstrated limited effectiveness as indicators of toxic heavy metal accumulation. The limited internal accumulation of toxic metals suggests possible physiological regulation or tolerance, although the long-term ecological effects require further investigation. Further controlled studies are needed before confirming their suitability for soil bioremediation, waste management, and vermicompost production.

## 1. Introduction

The heavy metal (HM) soil pollution disturbs soil fauna activity which effects ecosystem functions qualitatively and quantitatively [1]. Heavy metals bioaccumulate and transfer through the food chains due to their persistent nature [2]. The heavy metal soil pollution studies are quite important as these are non-biodegradable and pose risk to public health [3,4]. Soil biota helps to peep through ecosystem health as these are useful bioindicators of pollution. There is a correlation between the concentration of pollutants in the soil and the tissues of biota [4].

Earthworms comprise, approximately, 80% of the total soil fauna in soil and various birds and vertebrates feed upon them [5,6]. Earthworms are ecosystem engineers and early colonizers of the terrestrial ecosystem. These are directly exposed to soil pollutants. Earthworms can survive in highly heavy metal polluted soils and are able to survive in extremely unfavorable conditions [7,8,9,10].

Earthworms are able to remove heavy metals from humus via their digestive system. They bioaccumulate these heavy metals inside their bodies instead of excreting them [11]. Earthworms accumulate various heavy metals from soil due to their close external and internal contact with the soil [12,13,14]. Soil physicochemical properties, heavy metal concentration and other environmental factors, such as soil moisture and temperature, have an impact on heavy metals [15].

Soil heavy metals enter the human body orally and through dermal contact and inhalation. The heavy metal contaminated soils have adverse effects on human health especially in children due to hand-to-mouth contact [16]. There are very limited studies regarding the role of earthworms in the bioaccumulation of heavy metals that recognize the prominent role of earthworms in the bioremediation of soil [17]. The role of earthworms in the bioaccumulation of heavy metals for soil remediation is highly recommended, being very cheap, when compared with other recent technologies, i.e., land filling, incineration, nitrification, soil flushing, soil washing, biological treatment and chemical treatment. The use of earthworms to remove and degrade toxins, referred to as vermiremediation, is quite a promising strategy to remove soil toxins [18,19]. The vermiremediation has been studied for more than 40 years, but it has just been recognized significantly [20]. It is hypothesized that earthworms can be used for soil remediation from heavy metal pollution and there is a strong relationship between the bioaccumulation factor (BAF) concentration of the heavy metals and the physicochemical parameter. There are fragmentary studies regarding the heavy metal bioaccumulation in Punjab. This is the first dissectional assessment across Punjab about the heavy metal bioaccumulation in earthworms. The aim of this study is to identify the potential of earthworms for the bioremediation of heavy metal pollution. The future direction is to use the potent populations of earthworms for waste management.

## 2. Materials and Methods

### 2.1. Study Area

This study was conducted in the Punjab province of Pakistan which is located in the central-eastern region of the country. It is the second-largest province of Pakistan by land area and the largest province by population. It covers an area of 205,344 km^2^, 31.1471° N, 75.3412° E, and is located at an altitude of 300 m above the sea level. The population of Punjab is 127,474,000 and it has a density 620/km^2^ (Figure S1).

### 2.2. Sample Collection and Chemical Analysis

#### 2.2.1. Sample Collection

**Figure 1:**
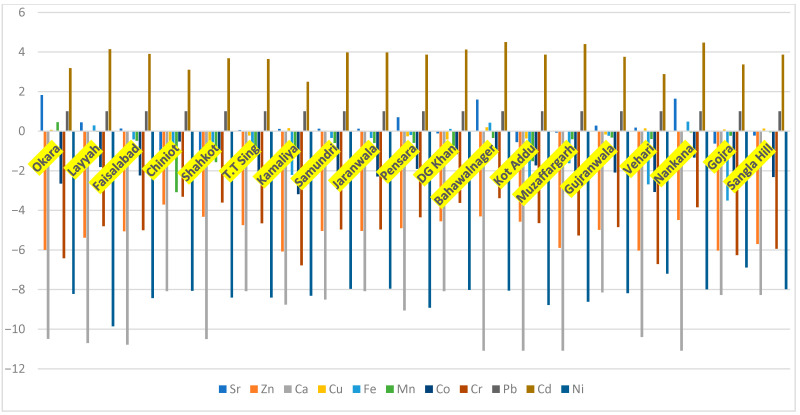
The pollution index for heavy metals in Punjab, Pakistan.

Nineteen sampling locations were selected across central and southern Punjab, Pakistan, namely: Okara, Layyah, Faisalabad, Chiniot, Shahkot, Toba Tek Singh, Kamaliya, Sammundri, Jaranwala, Pensara, Dera Ghazi Khan, Bahawalnagar, Kot Addu, Muzaffargarh, Gujranwala, Vehari, Nankana Sahib, Gojra, and Sangla Hill (Figure 1). These sites were selected using a random lottery method to represent agricultural and peri-urban soils of Punjab. Soil and earthworm samples were collected from each location during September 2022 to February 2023. Approximately 500 g soil with earthworms was sampled using scoop method and collected with zipper bags. The soil samples were dried, ground, and stored in bottles, in labs, for further chemical analysis. The earthworms were sorted out from the soil for further processing. All the samples were labeled with name, collector, site, and date. The soil and earthworms were proceeding for further chemical analysis [21].

#### 2.2.2. Sample Preparation and Analytical Method

Dried and homogenized soil and earthworm samples from the 19 locations were digested separately following a modified protocol of Uddin et al. (2016) [22]. The worms were analyzed whole (including gut contents). All digestion flasks were soaked in 10% HNO_3_ for 24 h, rinsed with Milli-Q water, dried, and labeled with sample IDs and blanks. Approximately 2 g of each soil sample and 2 g (fresh weight equivalent) of earthworm tissue were weighed into the flasks, and a few glass beads were added as anti-bumping granules. Then, 20 mL of concentrated HNO_3_ (65%) and 10 mL of HClO_4_ were added to each flask. The samples were allowed to pre-digest on a shaker overnight at room temperature and subsequently heated on a hot plate at about 120 °C for ~3 h until the brown fumes disappeared and a clear solution of approximately 5–10 mL remained. After cooling, 30 mL of deionized water was added, the mixture was filtered, and the residue was rinsed with an additional 30 mL of deionized water. The combined filtrate was collected in acid-washed volumetric flasks and used for instrumental analysis. Concentrations of Sr, Zn, Ca, Cu, Fe, Mn, Co, Cr, Pb, Cd, and Ni were determined by inductively coupled plasma mass spectrometry (ICP-MS; Prodigy 7, Teledyne Leeman Labs, Government College University Faisalabad, Pakistan). All the chemicals were purchased from Sigma-Aldrich (St. Louis, MO, USA).

#### 2.2.3. Quality Control and Analytical Method

To ensure analytical accuracy and precision, a multi-element reference standard solution with known concentrations of Sr, Zn, Ca, Cu, Fe, Mn, Co, Cr, Pb, Cd and Ni was analyzed after every 10 samples on the ICP-MS. This quality control standard was used to monitor instrument stability and to calculate analytical recoveries for all metals. All chemicals were of analytical grade. All plasticware and glassware were soaked in 10% HNO_3_ for 24 h and then rinsed twice with double-distilled deionized water before use.

### 2.3. Calculation of Pollution Level

#### Soil Geoaccumulation Index (Igeo) Method

The soil geoaccumulation index (Igeo) method was applied to assess heavy metal concentrations in the sampled areas of Punjab [23]. Following Aziz et al. (2023), this method was used to evaluate the degree of heavy metal pollution in the soils of the study area [23]. Soil quality was classified according to standard pollution level categories (Table S1). The basic calculation model is described below:

Igeo = log2 (C/1.5B)

Here, C represents the concentration of heavy metals (mg/kg), and B is the background value of the elements (Table S2).

### 2.4. Bioaccumulation Factor

The bioaccumulation factor (BAF) explains the concentration of heavy metal in the medium (soil) and the body of organisms (earthworm) [24]. It shows the ability of the organisms to uptake the strontium, zinc, calcium, copper, iron, manganese, cobalt, chromium, lead, cadmium, and nickel from the environment and is the most common way to study the behavior of these heavy metals as a bioaccumulant [25]. The calculation is expressed as follows:

BAFs = Conc._(body)_/Conc._(soil)_

where conc._(body)_ is the concentration of the heavy metal in the body and conc._(soil)_ is the concentration of heavy metal in the soil from where the earthworm was sampled.

### 2.5. Relationship Between Bioaccumulation Factor and Abiotic Factors Through Regression Analysis and Cluster Analysis

#### 2.5.1. Regression Analysis

Linear regression model was constructed to assess the relationship between bioaccumulation factors of heavy metals such as strontium, zinc, calcium, copper, iron, manganese, cobalt, chromium, lead, cadmium, and nickel as dependent variables, and various abiotic factors such as moisture, pH, electrical conductivity, total suspended solids, and total dissolve solids as independent variables following. Similarly, the relationship between heavy metal concentration in soil and bioaccumulation of the other heavy metals was studied.

#### 2.5.2. Cluster Analysis

Cluster analysis was carried out to investigate the degree of association or resemblance of the heavy metal bioaccumulation of strontium, zinc, calcium, copper, iron, manganese, cobalt, chromium, lead, cadmium, and nickel and heavy metal bioaccumulation in different regions [26].

## 3. Results

### 3.1. Pollution Level of Heavy Metals

Among all the other heavy metals, the Cd was found in most critical concentrations in almost all studied districts. Most of the locations showed intense to strong Cd pollution (Igeo > 3). For example, the five districts, Layyah, Dera Ghazi Khan, Bahawalnagar, Muzaffargarh, and Nankana, showed very high contamination levels of Cd (Igeo > 4). Mild to moderate pollution levels of lead (Pb) and strontium (Sr) were observed in different districts, with Igeo values ranging from 0 to 1. Lead (Pb) contamination was observed in agricultural and industrial regions, whereas strontium (Sr) contamination showed spatial variability. Moderate levels of Sr were found in Bahawalnagar, Nankana, and several other urban-adjacent districts. Similarly, the three heavy metals, copper (Cu), manganese (Mn), and iron (Fe), showed mild pollution in industrial and intensively cultivated areas. In contrast, zinc (Zn), calcium (Ca), chromium (Cr), cobalt (Co), and nickel (Ni) generally exhibited negative Igeo values, indicating unpolluted soil conditions across the majority of districts. Overall, the results demonstrate a region-wide dominance of cadmium contamination, with secondary contributions from Pb and Sr, while most other metals remained within natural background levels (Figure 1).

### 3.2. Cluster Analysis

#### 3.2.1. Cluster Analysis of Bioaccumulation Factor Across Central Punjab, Pakistan

**Figure 2:**
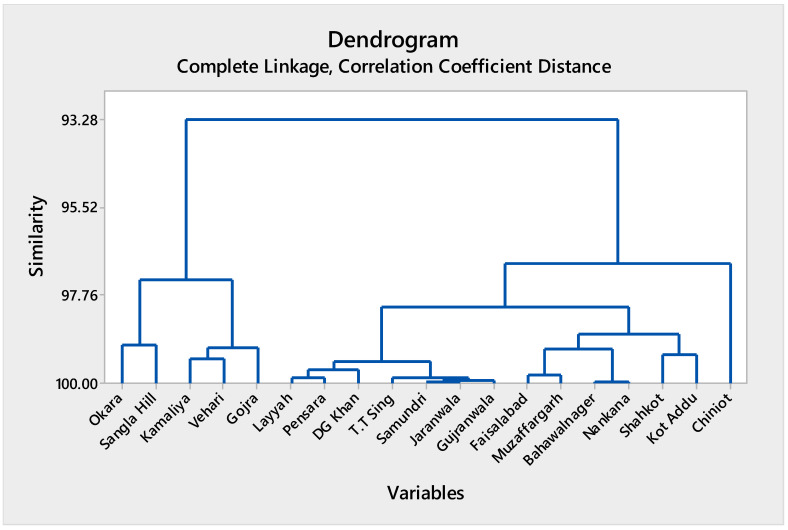
Cluster of observations of bioaccumulation factors in different regions of Central Punjab, Pakistan.

A high degree of similarity among observations was revealed by the hierarchical cluster analysis of bioaccumulation factors, with most clusters merging at similarity levels greater than 95% (Table S3; Figure 2). This clustering pattern indicates strong homogeneity in bioaccumulation behavior across the soils of the studied region. Several compact clusters formed at very high similarity levels (>99%), suggesting that many sampling locations share nearly identical bioaccumulation characteristics. As clustering progressed, larger groups merged gradually, with the final aggregation occurring at a similarity level above 93%, indicating limited spatial variability in overall bioaccumulation patterns. Overall, the dendrogram structure demonstrates that bioaccumulation factors across Central Punjab are regionally consistent, likely reflecting comparable environmental exposure conditions and soil–plant interactions (Table S3; Figure 2).

#### 3.2.2. Cluster Analysis of Heavy Metal Concentrations in Soil of Central Punjab, Pakistan

**Figure 3:**
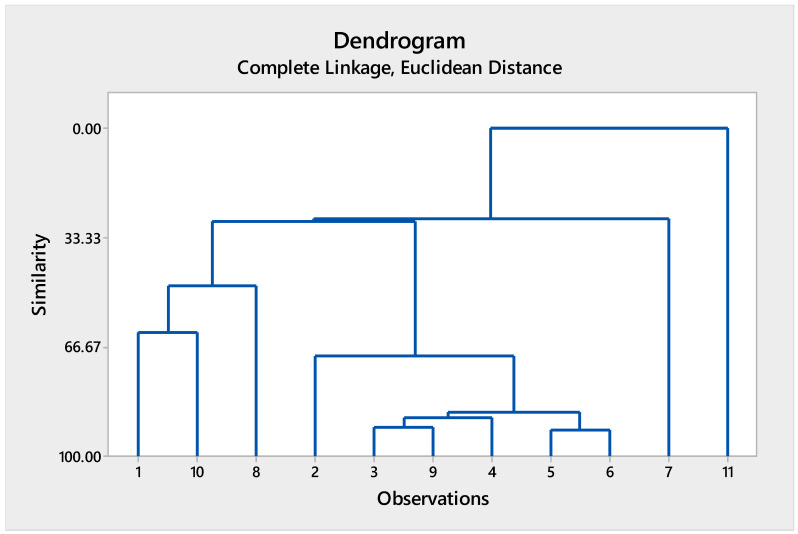
Cluster of observations of heavy metal concentration in soil in different regions of Central Punjab, Pakistan. Here, 1 = strontium, 2 = zinc, 3 = calcium, 4 = copper, 5 = iron, 6 = manganese, 7 = cobalt, 8 = chromium, 9 = lead, 10 = cadmium, and 11 = nickel.

The analyzed heavy metals were grouped based on their concentrations and geochemical behavior, showing distinct clustering patterns (Table S4; Figure 3). Several metals formed strongly associated clusters at high similarity levels (>85%), indicating shared sources or similar distribution mechanisms in the soil environment. In contrast, cadmium separated from other metals at lower similarity levels, highlighting its distinct contamination pattern and greater variability across the study region. The metals such as iron, manganese, and copper showed closer associations, suggesting natural background control or mixed lithogenic–anthropogenic inputs. The overall cluster structure supports the dominance of cadmium as a key contaminant in Central Punjab soils, while most other metals exhibited comparatively uniform spatial distributions.

### 3.3. Bioaccumulation of Heavy Metals in Earthworms and Total Soil Content

A bioaccumulation factor (BAF) greater than 1 indicates preferential accumulation of metals in earthworms relative to the soil. In the current study, earthworms exhibited significant bioaccumulation of Ca, Mn, Cu, and Sr, whereas Zn, Fe, Co, Cr, Pb, and Cd generally showed BAF values below one (Table 1). Among all analyzed elements, Ca showed the highest bioaccumulation potential, followed by Mn, Cu, and Sr, indicating strong physiological uptake and retention by earthworms. In contrast, group-1 carcinogenic metals, particularly lead, cadmium, and chromium, did not exhibit consistent bioaccumulation, suggesting limited biological uptake despite their presence in soil. Mean BAF values across Punjab followed the order Ca > Mn > Cu ≈ Sr > Ni > Zn > Co > Cr > Pb > Cd > Fe, highlighting clear differences in metal bioavailability and earthworm accumulation behavior.

Spatial variability in BAF values was observed across districts, with some locations exhibiting higher levels of accumulation for specific metals; however, these variations did not alter the overall regional bioaccumulation trend (Table 1). Collectively, the results indicate that earthworms preferentially accumulate essential and mobile elements, while toxic metals such as cadmium and lead show limited bioaccumulation.

Linear regression analysis revealed very weak relationships between soil metal concentrations and bioaccumulation factors (BAFs) in earthworms across all analyzed elements (Table 2).

The coefficients of determination (R^2^) were consistently low, ranging from 0.0001 to 0.05, indicating that soil metal concentration explained only a small proportion of the variation in earthworm bioaccumulation. Slight positive relationships were observed for calcium, copper, cobalt, chromium, lead, cadmium, and nickel, whereas negative trends were found for strontium, iron, and manganese. However, none of these relationships showed strong predictive power. Overall, the results show that bioaccumulation in earthworms is not solely controlled by heavy metal concentrations in the soil. Other factors, such as metal bioavailability, physicochemical properties of the soil, and physiological regulation, also play important roles in bioaccumulation (Table 2).

### 3.4. Impacts of Abiotic Factors on the Bioaccumulation of Heavy Metals

Regression analysis indicated that abiotic soil variables (moisture, pH, electrical conductivity, total suspended solids, and total dissolved solids) had generally weak relationships with the BAF of metals in earthworms (Table S5). Across most metals and predictors, the coefficients of determination (R^2^) were very low (typically < 0.05), indicating that these factors explained only a small proportion of variation in bioaccumulation.

Among the evaluated predictors, total dissolved solids (TDS) and electrical conductivity (EC) showed the relatively strongest associations with bioaccumulation, particularly for manganese (TDS: R^2^ = 0.139; EC: R^2^ = 0.099) and copper (TDS: R^2^ = 0.124). Moderate relationships were also observed between strontium and EC (R^2^ = 0.103) and between iron and EC (R^2^ = 0.089). In contrast, total suspended solids (TSS) and pH showed minimal explanatory power for most metals. Overall, the findings suggest that no single abiotic factor strongly predicts earthworm bioaccumulation, and that bioaccumulation is likely driven by a combination of soil chemistry and metal-specific biological regulation (Table S5).

### 3.5. Relationship Between Metal Concentration in Soil and Bioaccumulation of Heavy Metals in Earthworms

Our results showed weak to moderate relationships between soil heavy metal concentrations and the bioaccumulation factors (BAFs) of earthworms (Table S6). The coefficients of determination (R^2^) indicated that soil metal concentrations explained only a limited proportion of the variation in earthworm bioaccumulation. However, several relationships showed comparatively higher explanatory power, suggesting coordinated behavior among certain metals. The strongest associations were observed for Cr as a predictor of BAF for multiple metals, particularly copper (R^2^ = 0.615) and cobalt (R^2^ = 0.675), along with manganese (R^2^ = 0.424). Similarly, Co concentration in soil showed strong relationships with copper bioaccumulation (R^2^ = 0.715) and manganese bioaccumulation (R^2^ = 0.534). In addition, zinc concentration was strongly associated with copper bioaccumulation (R^2^ = 0.642) and manganese bioaccumulation (R^2^ = 0.603). For the BAFs of Sr and Cu, Mn showed moderate relationships, with R^2^ values of 0.328 and 0.471, respectively. In contrast, the relationships involving Pb, Cd, and Ni were generally weak, indicating that their bioaccumulation is not well predicted by overall soil contamination levels (Table S6).

## 4. Discussion

Heavy metal activity in soil varies greatly between countries and is dependent on the type and amount of heavy metal [27,28]. The chemical and mineralogical nature of metal pollutants from the emission source and physicochemical properties of the recipient soil environments determine the bioavailability and metal distribution [29]. The soil quality is considered to be clean if it has an Igeo value of zero or less than zero [24]. The chelation of heavy metals with natural organic substances makes them less bioavailable in soil [30]. Our results are in clear contrast to the findings of Dai et al. (2004) [31], who reported the bioaccumulation factor (BAF) pattern as Cd > Zn > Cu > Pb. In their study, the mean BAF values showed a wide range across metals, with cadmium concentrations ranging from 6.18 to 17.02, zinc from 1.95 to 7.91, copper from 0.27 to 0.89, and lead from 0.08 to 0.38, indicating very high BAFs and comparatively broader variability [31].

The presence of heavy metals in the soil and groundwater are responsible for the absorption in plants and other biotic fauna including humans [32]. Our results indicate excessive levels of Cd in the soils of almost all districts. Due to its high mobility, the likelihood of heavy metal transfer into living organisms is greater. Furthermore, cadmium exhibits higher mobility in both soil and water compared to other metals. The order of metal mobility already observed in the previous study is as follows: Cd > Ni > Zn > Mn > Cu > Pb = Cr [33]. Further studies confirm that Cd has higher mobility, and a strong relationship has been reported between Cd concentrations in soil and its accumulation in fruits, vegetables, and crops such as rice [34]. As a pollutant, cadmium can accumulate in chloroplasts and disrupt chloroplast processes in crops such as maize and barley [35]. Not only the plants, the other invertebrates such as earthworms, isopods, and gastropods in the soil food web are at risk of Cd accumulation and biomagnification [36]. However, excessive amounts of Fe, Mn, Zn, and Cu may have an impact on *Legionella pneumophila* and *Mycobacterium tuberculosis* resistance [18,37]. The BAF in *N. calginosus* is Cd (11.2) > Zn (5.9) > Cu (0.49) > Pb (0.03) [13]. Long-term heavy metal exposure to earthworms has a negative effect on the survival, density, growth, behavior, sexual development and cocoon production [38]. The heavy metals, i.e., Pb, Zn, Hg, Cu, and Cd, bioaccumulate in the earthworms and have extremely negative impacts on them by reducing their reproductive potential, biomass, cell division and the activities of the antioxidant enzyme. They also damage DNA and nephridia [39,40]. The earthworms bioaccumulate cadmium much more than lead [41] which is in contrast to our study. The bioaccumulation of cadmium in earthworms is through the intestines during digestion and through dermal absorption from soil solution. The water-soluble metal salts add to the efficient uptake of metals through the skin. The cadmium uptake by the earthworms is not affected by the soil pH [42,43].

There is a positive relationship between zinc concentration in soil and its bioaccumulation in earthworms [44]; however, the relationship is not significant [45]. There is a positive relationship between copper concentration in soil and its BAF in earthworms [44]; however, the BAF of copper is positively related to the cadmium concentration in soil [45]. There is a positive relationship between the BAF of lead concentration and its concentration in soil [44]. The results are in uniformity with the findings of Vinodhini et al. (2008) showing that lead concentration was significantly increased in the tissues [38]. There is a positive relationship between cadmium concentration in soil and earthworms [44]. The cadmium concentration in *L. rubellus* was positively correlated to that of soil [45]. The cadmium concentration significantly increased in the tissues of *Cyprinus carpio* (common carp) and relates positively with that of water concentration [38].

The amount of metal accumulated in earthworm tissues partially depends on the absolute concentration of the metal in a particular soil; however, physiochemical and edaphic interactions, including variables like pH, calcium concentration, organic matter content, C-to-N ratio, and cation-exchange capacity, strongly influence this process [39,40,41,46]. Additionally, it has been proposed that inter-specific variations in nutritional metal intakes and physiological utilization also influence the patterns of metal accumulation in earthworm tissues [40,42]. Heavy metals may accumulate in their tissues as a result of their feeding activities, which may restrict their participation in the food chain by affecting the availability or total heavy metal concentrations [43,47].

The relationship between the bioaccumulation of heavy metals in the earthworms and the physicochemical parameters gives an insight regarding environmental manipulation for the development of effective strategies for environmental bioremediation. This technique is an unsupervised classification procedure that involves a measurement of the similarity between objects to be clustered [47]. The heavy metals that show similar trends of bioaccumulation in earthworms are shown through cluster analysis. Gastropods such as Physids have shown strong bioaccumulation potential of chromium which is a group-1 carcinogenic heavy metal [23]. The gastropods are seen more from the collection sites as compared to the earthworms (personal communication) which might be due to the decrease in the microbial populations due to the heavy metal pollution, thereby leaving more debris for the gastropods in comparison to the amount of humus for the earthworms. Similarly, the ant population has a positive relationship with the heavy metal concentration [21,48]. The sensitivity of earthworms towards heavy metal pollution needs to be further studied through lab cultures and if these are found to be sensitive then the population dynamics of the earthworms and gastropods will explain the impact of the heavy metal pollution on the invertebrate; however, if these are found to be insensitive then these can be used for the management of waste from different sources, i.e., domestic, agriculture and hospital sources.

## 5. Conclusions

This study concludes that several regions of Punjab, Pakistan, exhibit strong to extreme soil contamination due to Cd. In contrast, most other heavy metals were found within the unpolluted to moderately polluted categories. BAF values showed high similarity among certain locations, particularly Samundri, Jaranwala, and Gujranwala, and strong consistency was also observed for elements such as Ca, Cu, Fe, and Mn. Among the measured soil properties, electrical conductivity showed a comparatively greater influence on BAF values than other parameters, followed by soil moisture and total dissolved solids; however, all observed relationships between bioaccumulation and soil physicochemical characteristics remained weak overall. Earthworms did not exhibit significant bioaccumulation of group-1 carcinogenic metals such as lead, cadmium, and chromium, while they showed greater uptake of certain essential or less strictly regulated elements including calcium, manganese, copper, and strontium. The weak relationships between BAF values and soil metal concentrations or physicochemical parameters indicate that earthworms in this region have limited effectiveness as bioindicators of toxic heavy metal bioaccumulation. The relatively low internal accumulation of highly toxic metals suggests possible physiological regulation or tolerance mechanisms; however, this does not reduce the broader ecological and environmental risks associated with soil heavy metal contamination, particularly cadmium. Under the environmental conditions of this study, earthworms did not demonstrate strong potential for heavy metal-based soil bioremediation. Further controlled and long-term investigations are needed to better understand their ecological role and to evaluate their possible applications in soil quality remediation, sustainable waste management, and vermicompost production.

## Figures and Tables

**Table 1:** Bioaccumulation factor (BAF) values [mean (min–max)] as the ratio between heavy element concentrations in earthworms and total soil contents.

Class		Sr	Zn	Ca	Cu	Fe	Mn	Co	Cr	Pb	Cd	Ni
Oligochaeta	Mean	1.24	0.75	2.01	1.24	0.22	1.28	0.39	0.34	0.23	0.28	1.71
	Range	(0.0048–6.82)	(0.18–3.83)	(0.15–8.18)	(0.41–2.72)	(0.00–1.13)	(0.0098–6.29)	(0.11–1.31)	(0.05–2.45)	(−0.45–1.06)	(0.07–1.31)	(−2.58–6.88)

**Table 2:** Linear regression relationships between soil metal concentrations and bioaccumulation factors (BAFs) in earthworms, showing regression equations and coefficients of determination (R^2^).

Heavy Metal	Regression Equation	R^2^
Strontium	Y = −0.1581x + 4199.8	0.0168
Zinc	y = 0.007x + 0.6368	0.0001
Calcium	y = 0.1092x + 0.038	0.02
Copper	y = 0.0289x + 32.486	0.001
Iron	y = −0.0421x + 1200.6	0.015
Manganese	y = −0.1252x − 547.2	0.05
Cobalt	y = 0.0105x + 1.7156	0.01
Chromium	y = 0.0134x + 1.0694	0.01
Lead	y = 0.1009x + 0.1196	0.03
Cadmium	y = 23.614x + 267.12	0.001
Nickle	y = 0.4763x + 0.5259	0.02

## Data Availability

All the data generated or analyzed during this study are included in the published article.

## Supplementary Materials

The following supporting information can be downloaded at: https://www.mdpi.com/article/10.3390/biology15040306/s1, Figure S1: Study Area Punjab, Pakistan (Saleem et al., 2014 [49]). Table S1: a: The pollution index classification standard and trace elements. b: The pollution index classification standard and trace elements for Strontium, Zinc, Calcium, Copper, Iron, Manganese, Cobalt, Chromium, Lead, Cadmium, and Nickle pollution degree classification. Table S2: Background value of heavy metal in soil. Table S3: Cluster of observations bioaccumulation factor in different region of Central Punjab, Pakistan. Table S4: Cluster of observation of Strontium, Zinc, Copper, Iron, Manganese, Cobalt, Chromium, Lead, Cadmium, and Nickel concentration in soil in different region of Central Punjab, Pakistan. Table S5: Impact of abiotic factors on the bioaccumulation of heavy metals. Table S6: Relationship between Metal Concentration in Soil and Bioaccumulation of Heavy metals in Earthworms.
