# Supplementary material for: Heavy Metal Pollution and Assessment of the Bioaccumulation Potential of Earthworms from the Soil of Punjab, Pakistan
Source: Biology (Basel). 2026 Feb 9;15(4):306. doi: 10.3390/biology15040306 (PMC12937666; doi:10.3390/biology15040306)
Supplement: Supplementary file 1 [file biology-15-00306-s001.zip › biology-4083831-supplementary.pdf]

Supplementary Material

Calculation of Pollution Level
Soil Geo-accumulation Index Method

$$I_{geo} = log_2\ (C/1.5B)$$

Where C is the measured concentration of trace elements and B is the background value of the corresponding elements. The back ground value for Arsenic is Where C is the measured concentration of trace elements, and B is the background value of the Arsenic is 19.05 mg/kg.

Weighted arithmetic Water Quality Index (WQI) method

$$q_i = C_i/S_i X100$$

$$W_i = 1/S_i$$

$$WQI = \sum q_i w_i / \sum w_i$$

Bioaccumulation factor

$$BAFs = C_{sp}\ /C_{medium}$$

Where C_{sp} is the concentration of heavy metals in oligocheats and C medium is the concentration of heavy metals in soil.

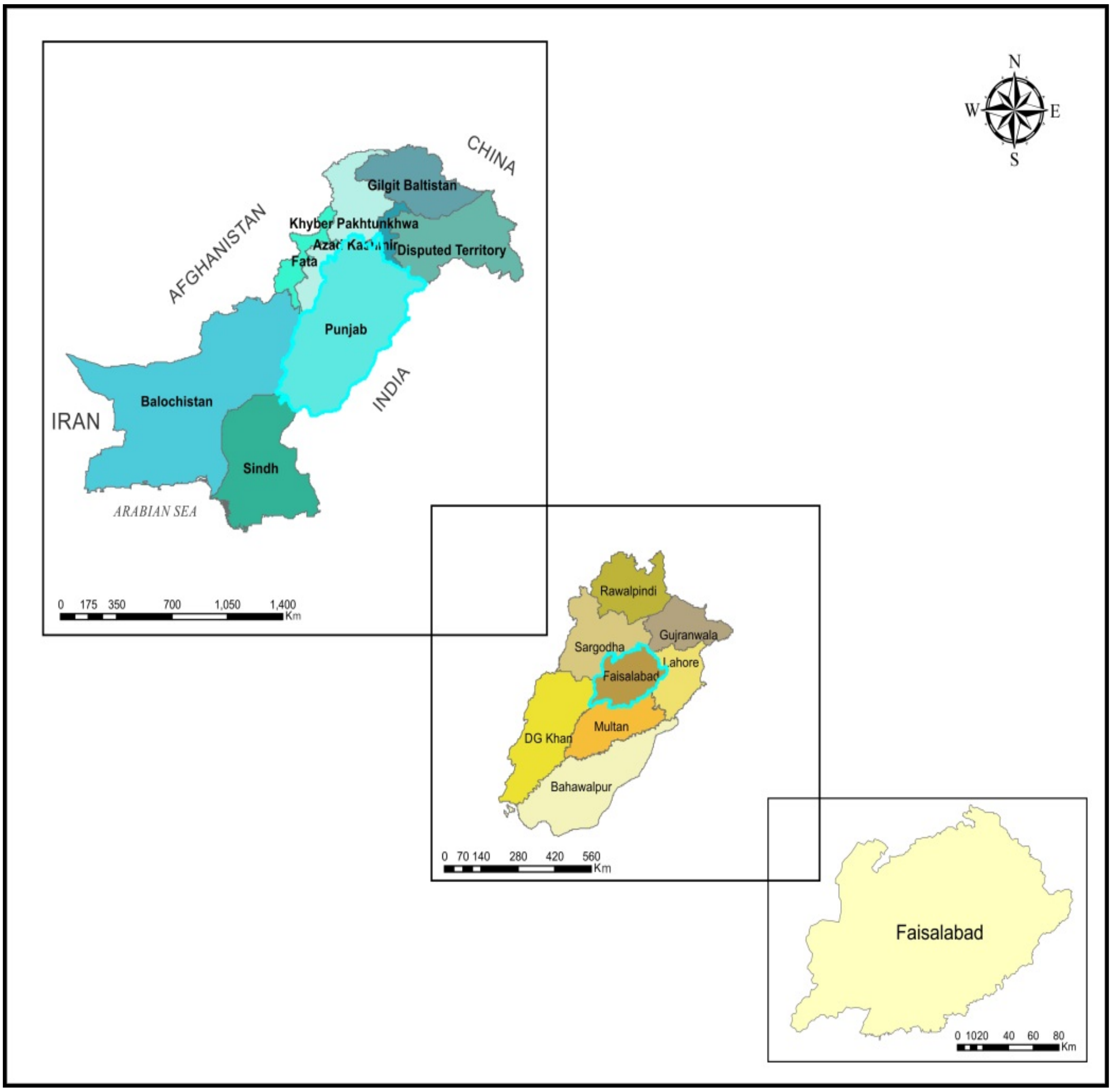

Figure S1: Study Area Punjab, Pakistan (Saleem et al., 2014).

Table S1a: The pollution index classification standard and trace elements

Class	Igeo	Pollution Level	Mean value
0	≤0	Clean	-
1	0<Igeo≤1	Mild-moderate	-
2	1<Igeo≤2	Moderate	-
3	2<Igeo≤3	Moderate-intense	-
4	3<Igeo≤4	Intense	-
5	4<Igeo≤5	Intense-strong	-
6	5<Igeo≤10	Very strong	-

Table S1b: The pollution index classification standard and trace elements for Strontium, Zinc, Calcium, Copper, Iron, Manganese, Cobalt, Chromium, Lead, Cadmium, and Nickle pollution degree classification.

Regions/Metals	Sr	Zn	Ca	Cu	Fe	Mn	Co	Cr	Pb	Cd	Ni
Okara	1.82	-5.99	-10.49	0.07	-0.01	0.45	-2.65	-6.42	1	3.18	-8.22
Layyah	0.44	-5.38	-10.7	-0.01	0.29	-0.04	-1.81	-4.8	1	4.14	-9.86
Faisalabad	0.13	-5.06	-10.78	-0.03	-0.41	-0.49	-2.23	-5	1	3.9	-8.43
Chiniot	-1.01	-3.7	-8.08	-1.29	-1.04	-3.08	-0.52	-3.31	1	3.1	-8.06
Shahkot	-1.28	-4.32	-10.5	-0.66	-0.56	-1.56	-1.04	-3.6	1	3.68	-8.4
T.T Sing	0.06	-4.74	-8.08	-0.22	-0.85	-0.81	-2.05	-4.65	1	3.64	-8.4
Kamaliya	0.11	-6.08	-8.76	0.15	-2.21	-0.93	-3.18	-6.78	1	2.49	-8.31
Samundri	0.12	-5.04	-8.5	0.01	-0.34	-0.6	-2.29	-4.96	1	3.97	-7.96
Jaranwala	0.12	-5.04	-8.08	0.01	-0.34	-0.6	-2.29	-4.96	1	3.97	-7.95
Pensara	0.7	-4.9	-9.06	-0.26	-0.19	-0.59	-1.9	-4.35	1	3.86	-8.92
DG Khan	-0.11	-4.55	-8.08	-0.41	0.11	-0.73	-1.16	-3.62	1	4.12	-8.02
Bahawalnager	1.59	-4.3	-11.08	0.19	0.42	-0.34	-1	-3.38	1	4.5	-8.05
Kot Addu	-0.55	-4.57	-11.08	-0.37	-2.57	-1.52	-1.73	-4.64	1	3.86	-8.78
Muzaffargarh	-0.07	-5.89	-11.08	-0.02	-1.62	-0.4	-2.34	-5.27	1	4.4	-8.61
Gujranwala	0.28	-4.98	-8.15	-0.16	-0.23	-0.32	-2.09	-4.84	1	3.75	-8.18
Vehari	0.18	-6.03	-10.4	0.14	-2.68	-0.39	-3.07	-6.71	1	2.88	-7.2
Nankana	1.64	-4.49	-11.08	-0.02	0.48	-0.06	-1.33	-3.84	1	4.47	-7.99
Gojra	-0.62	-6.03	-8.27	0.08	-3.5	-0.23	-2.9	-6.26	1	3.37	-6.89
Sangla Hill	-0.22	-5.7	-8.27	0.13	-0.03	-0.05	-2.32	-5.94	1	3.86	-7.98

Table S2: Background value of heavy metal in soil

Sr. no	Heavy metal	Background value (mg/kg)
1	Sr	2237
2	Zn	21
3	Cd	3
4	Pb	22
5	Cr	88
6	Co	13
7	Ni	72
8	Mn	654
9	Fe	5357
10	Cu	23
11	Ca	14.44

Table S3: Cluster of observations bioaccumulation factor in different region of Central Punjab, Pakistan

step		No of cluster	Similarity level	Distance level	Cluster joined		New cluster	No. of observation in new cluster
1		18	99.8910	0.000380	8	9	8	2
2		17	99.9481	0.001039	12	17	12	2
3		16	99.9411	0.001178	8	15	8	3
4		15	99.8615	0.002770	2	10	2	2
5		14	99.8528	0.002943	6	8	6	4
6		13	99.7750	0.004499	3	14	3	2
7		12	99.6447	0.007105	2	11	2	3
8		11	99.4575	0.010849	2	6	2	7
9		10	99.3648	0.012703	7	16	7	2
10		9	99.2716	0.014568	5	13	5	2
11		8	99.1417	0.017167	3	12	3	4
12		7	99.1118	0.017765	7	18	7	3
13		6	99.0423	0.019153	1	19	1	2
14		5	98.7502	0.024995	3	5	3	6
15		4	89.0660	0.038679	2	3	2	13
16		3	97.3663	0.052674	1	7	1	5
17		2	96.9391	0.061217	2	4	2	14
18		1	93.2786	0.134428	1	2	1	19

Table S4: Cluster of observation of Strontium, Zinc, Copper, Iron, Manganese, Cobalt, Chromium, Lead, Cadmium, and Nickel concentration in soil in different region of Central Punjab, Pakistan

step	No of cluster	Similarity level	Distance level	Cluster joined		New cluster	No of observation in new cluster
1	10	91.9185	1.9246	5	6	5	2
2	9	91.3724	2.0546	3	9	3	2
3	9	88.3319	2.7787	3	4	3	3
4	7	86.4938	3.2164	3	5	3	5
5	6	69.2604	7.3205	2	3	2	6
6	5	62.0555	9.0363	1	10	1	2
7	4	47.8980	12.4078	1	8	1	3
8	3	28.4057	17.0499	1	2	1	9
9	2	27.4432	17.2791	1	7	1	10
10	1	0.0000	23.8145	1	11	1	11

Table S5: Impact of abiotic factors on the bioaccumulation of heavy metals

Heavy Metals	Abiotic factors	Regression equation	R^2
Strontium	Moisture	y=0.057x-0.0496	0.0513
	pH	y=-0.4145x+4.001	0.0596
	EC	y=-0.0008x+1.611	0.1033
	TSS	y=-0.0031x+1.3581	0.0004
	TDS mg/g	y=-0.0019x+1.6144	0.0676
Zinc	Moisture	y=-0.0035x+0.8284	0.0006
	pH	y=0.102x+0.0691	0.0107
	EC	y=-0.0001x+0.8084	0.0077
	TSS	y=0.0101x+0.3562	0.0126
	TDS mg/g	y=0.0003x+0.684	0.006
Calcium	Moisture	y=-0.07x+3.5871	0.0246
	pH	y=0.0513x+1.6642	0.0003

	EC	y=-0.001x+2.4497	0.0466
	TSS	y=0.0195x1.246	0.0051
	TDS mg/g	y=-0.0009x+2.1851	0.0049
Copper	Moisture	y=0.0248x+0.6842	0.0465
	pH	y=-0.1728x+2.395	0.0499
	EC	y=-0.0002x+1.3235	0.023
	TSS	y=0.005x+1.0489	0.005
	TDS mg/g	y=-0.0012x+1.4756	0.1242
Iron	Moisture	y=-0.0066x+0.37	0.0184
	pH	y=0.0147x+0.1226	0.002
	EC	y=-0.0001x+0.2875	0.0886
	TSS	y=0.0019x+0.1472	0.004
	TDS mg/g	y=-0.0003x+0.2822	0.0483
Manganese	Moisture	y=0.092x-0.8012	0.0783
	pH	y=-0.3952x+3.9099	0.0318
	EC	y=-0.001x+1.7525	0.0993
	TSS	y=0.0339x-0.0489	0.0286
	TDS mg/g	y=-0.0035x+1.9799	0.1391
Cobalt	Moisture	y=-0.004x+0.4817	0.0073
	pH	y=0.0457x+0.0874	0.0212
	EC	Y=-0.00005x+0.4166	0.0136
	TSS	y=0.0044x+0.2186	0.0243
	TDS mg/g	y=0.0001x+0.3653	0.0097
Chromium	Moisture	y=-0.0038x+0.4225	0.0026
	pH	y=0.0538x-0.0222	0.0113
	EC	y=-0.000007x+0.339	8.00E-05
	TSS	y=0.0084x+0.008	0.0336
	TDS mg/g	y=0.0004x+0.3281	0.0003
Lead	Moisture	y=0.009x+0.0274	0.0254
	pH	y=-0.0119x+0.3103	0.001
	EC	y=-0.0001x+0.2822	0.0383
	TSS	y=0.0002x+0.02233	3E-0.5
	TDS mg/g	y=-0.00009x+0.2486	0.0029
Cadmium	Moisture	y=0.0047x+0.1773	0.0103
	pH	y=0.0039x+0.2574	0.0002
	EC	y=-0.0001x+0.3357	0.0596
	TSS	y=0.0062x+0.0434	0.047
	TDS mg/g	y=-0.0002x+0.3283	0.0282
Nickel	Moisture	y=-0.0542x+2.9303	0.0387
	pH	y=0.0822x+1.1591	0.002
	EC	y=0.0003x+1.5904	0.0085
	TSS	y=-0.0047x+1.8889	0.0008
	TDS mg/g	y=-0.0004x+1.7804	0.0021

Table S6: Relationship between Metal Concentration in Soil and Bioaccumulation of Heavy metals in Earthworms

Calcium	Regression Equation	R^2
Strontium	y = 10.395x + 0.8161	$R^2 = 0.0734$
Zinc	y = -4.5971x + 0.9358	$R^2 = 0.0424$
Copper	y = 7.035x + 0.9575	$R^2 = 0.1617$
Iron	y = -0.155x + 0.2268	$R^2 = 0.0004$
Manganese	y = 17.709x + 0.5565	$R^2 = 0.125$
Cobalt	y = -1.7202x + 0.4615	$R^2 = 0.0588$
Chromium	y = -1.8093x + 0.4096	$R^2 = 0.025$
Lead	y = -0.7379x + 0.2612	$R^2 = 0.0073$
Cadmium	y = 0.6172x + 0.2585	$R^2 = 0.0076$
Nickle	y = -6.5145x + 1.9718	$R^2 = 0.0241$
Iron	Regression Equation	R^2
Strontium	y = -0.0001x + 1.8773	$R^2 = 0.0794$
Zinc	y = 2E-06x + 0.7351	$R^2 = 0.0001$
Copper	y = -3E-05x + 1.4048	$R^2 = 0.0244$
Calcium	y = -0.0001x + 2.5894	$R^2 = 0.0211$
Manganese	y = -0.0002x + 2.1987	$R^2 = 0.0972$
Cobalt	y = -2E-05x + 0.5201	$R^2 = 0.0937$
Chromium	y = -1E-05x + 0.3993	$R^2 = 0.0087$
Lead	y = -9E-06x + 0.2815	$R^2 = 0.0097$
Cadmium	y = -3E-05x + 0.4583	$R^2 = 0.1744$
Nickle	y = -4E-05x + 1.9538	$R^2 = 0.0099$
Mangneses	Regression Equation	R^2
Strontium	y = 0.002x + 2.5353	$R^2 = 0.3282$
Zinc	y = -0.0008x + 0.2289	$R^2 = 0.156$
Copper	y = 0.0011x + 1.9513	$R^2 = 0.4707$
Calcium	y = -0.0006x + 1.5952	$R^2 = 0.0105$
Iron	y = 8E-05x + 0.274	$R^2 = 0.0148$
Cobalt	y = -0.0003x + 0.2099	$R^2 = 0.1885$
Chromium	y = -0.0004x + 0.0987	$R^2 = 0.1236$
Lead	y = 0.0001x + 0.322	$R^2 = 0.0318$
Cadmium	y = 0.0002x + 0.4032	$R^2 = 0.0821$
Nickle	y = 3E-05x + 1.7292	$R^2 = 8E-05$
Chromium	Regression Equation	R^2
Strontium	y = 0.1137x + 0.5259	$R^2 = 0.1804$
Zinc	y = -0.0818x + 1.2619	$R^2 = 0.2763$
Copper	y = 0.0957x + 0.6437	$R^2 = 0.6148$
Calcium	y = -0.0783x + 2.497	$R^2 = 0.0273$
Iron	y = -0.014x + 0.3086	$R^2 = 0.0732$
Cobalt	y = -0.0407x + 0.6465	$R^2 = 0.6751$
Manganese	y = 0.2275x - 0.15	$R^2 = 0.4241$
Lead	y = 0.0021x + 0.2179	$R^2 = 0.0012$
Cadmium	y = -1E-04x + 0.2841	$R^2 = 4E-06$
Nickle	y = 0.002x + 1.6949	$R^2 = 4E-05$
Strontium	Regression Equation	R^2

Zinc	y = 3E-05x + 0.6419	R^2 = 0.0121
Calcium	y = -2E-06x + 2.0134	6.00E-06
Copper	y = -8E-05x + 1.5519	R^2 = 0.164
Iron	y = -2E-05x + 0.2979	R^2 = 0.0536
Manganese	y = -0.0002x + 2.1722	R^2 = 0.1687
Cobalt	y = 1E-05x + 0.3307	R^2 = 0.0389
Chromium	y = 3E-05x + 0.2197	R^2 = 0.0544
Lead	y = -6E-06x + 0.2573	R^2 = 0.0048
Cadmium	y = -7E-06x + 0.3119	R^2 = 0.0084
Nickle	y = -3E-05x + 1.8242	R^2 = 0.0041
Cadmium	Regression Equation	R^2
Strontium	y = -0.0211x + 2.5516	R^2 = 0.1451
Zinc	y = 0.0005x + 0.7199	R^2 = 0.0002
Calcium	y = 0.0205x + 0.7276	R^2 = 0.0438
Copper	y = -0.0089x + 1.7993	R^2 = 0.1251
Iron	y = -0.0047x + 0.5107	R^2 = 0.1881
Manganese	y = -0.0328x + 3.3195	R^2 = 0.2061
Cobalt	y = -0.0017x + 0.4955	R^2 = 0.0265
Chromium	y = -0.0032x + 0.5341	R^2 = 0.0371
Lead	y = -0.0032x + 0.4294	R^2 = 0.0654
Nickle	y = 0.0027x + 1.536	R^2 = 0.0021
Lead	Regression Equation	R^2
Strontium	y = -0.5996x + 1.9501	R^2 = 0.0526
Zinc	y = 0.2812x + 0.4152	R^2 = 0.0342
Calcium	y = 0.8136x + 1.0407	R^2 = 0.0309
Copper	y = -0.1694x + 1.4444	R^2 = 0.0202
Iron	y = -0.1856x + 0.4408	R^2 = 0.134
Manganese	y = -0.6304x + 2.0241	R^2 = 0.0341
Cobalt	y = -0.0658x + 0.4697	R^2 = 0.0185
Chromium	y = -0.0797x + 0.4307	R^2 = 0.0105
Cadmium	y = -0.21x + 0.5328	R^2 = 0.1897
Nickle	y = 0.0746x + 1.6186	R^2 = 0.0007
Nickel	Regression Equation	R^2
Strontium	y = 0.3379x + 1.1175	R^2 = 0.0041
Zinc	y = -0.5419x + 0.9428	R^2 = 0.0311
Calcium	y = -1.528x + 2.5528	R^2 = 0.0267
Copper	y = 0.1523x + 1.1888	R^2 = 0.004
Iron	y = 0.2409x + 0.1344	R^2 = 0.0552
Manganese	y = 0.5332x + 1.0851	R^2 = 0.006
Cobalt	y = 0.042x + 0.3766	R^2 = 0.0019
Chromium	y = 0.0388x + 0.3222	R^2 = 0.0006
Cadmium	y = 0.207x + 0.2095	R^2 = 0.0451
Lead	y = 0.0423x + 0.2161	R^2 = 0.0013
Chromium	Regression Equation	R^2
Strontium	y = 0.1137x + 0.5259	R^2 = 0.1804
Zinc	y = -0.0818x + 1.2619	R^2 = 0.2763
Calcium	y = -0.0783x + 2.497	R^2 = 0.0273
Copper	y = 0.0957x + 0.6437	R^2 = 0.6148

Iron	y = -0.014x + 0.3086	R^2 = 0.0732
Manganese	y = 0.2275x - 0.15	R^2 = 0.4241
Cobalt	y = -0.0407x + 0.6465	R^2 = 0.6751
Cadmium	y = -1E-04x + 0.2841	R^2 = 4E-06
Lead	y = 0.0021x + 0.2179	R^2 = 0.0012
Nickle	y = 0.002x + 1.6949	R^2 = 4E-05
Cobalt	Regression Equation	R^2
Strontium	y = 0.1438x + 0.35	R^2 = 0.2188
Zinc	y = -0.0864x + 1.2829	R^2 = 0.2335
Calcium	y = -0.1128x + 2.7035	R^2 = 0.0429
Copper	y = 0.1185x + 0.5112	R^2 = 0.7147
Iron	y = -0.0133x + 0.3027	R^2 = 0.0497
Manganese	y = 0.2933x - 0.536	R^2 = 0.5341
Chromium	y = -0.0432x + 0.6028	R^2 = 0.2219
Cadmium	y = 0.0027x + 0.2668	R^2 = 0.0023
Lead	y = 0.0017x + 0.2206	R^2 = 0.0006
Nickle	y = 0.0041x + 1.682	R^2 = 0.0001
Zinc	Regression Equation	R^2
Strontium	y = 0.8152x + 0.286	R^2 = 0.2285
Cobalt	y = -0.2235x + 0.6527	R^2 = 0.502
Calcium	y = -0.2936x + 2.3495	R^2 = 0.0094
Copper	y = 0.623x + 0.5154	R^2 = 0.642
Iron	y = -0.0682x + 0.3001	R^2 = 0.0424
Manganese	y = 1.7288x - 0.7438	R^2 = 0.603
Chromium	y = -0.2362x + 0.612	R^2 = 0.2158
Cadmium	y = 0.0642x + 0.2085	R^2 = 0.0417
Lead	y = 0.0603x + 0.1608	R^2 = 0.0247
Nickle	y = -0.0614x + 1.7789	R^2 = 0.0011
